# Spatial variation in toll-like receptor diversity in koala populations across their geographic distribution

**DOI:** 10.1007/s00251-024-01365-5

**Published:** 2024-11-30

**Authors:** Jian Cui, Kimberley C. Batley, Luke W. Silver, Elspeth A. McLennan, Carolyn J. Hogg, Katherine Belov

**Affiliations:** 1 School of Life and Environmental Sciences, The University of Sydney, Sydney, NSW 2006 Australia; 2 ARC Centre of Excellence for Innovations in Peptide and Protein Science, The University of Sydney, Sydney, NSW Australia

**Keywords:** Immune genes, Single-nucleotide polymorphism, Whole-genome sequences, Conservation genomics, Diseases

## Abstract

The koala (*Phascolarctos cinereus*) is an iconic Australian species that is listed as endangered in the northern parts of its range due to loss of habitat, disease, and road deaths. Diseases contribute significantly to the decline of koala populations, primarily *Chlamydia* and koala retrovirus. The distribution of these diseases across the species’ range, however, is not even. Toll-like receptors (TLRs) play a crucial role in innate immunity by recognising and responding to various pathogens. Variations in TLR genes can influence an individual’s susceptibility or resistance to infectious diseases. The aim of this study was to identify koala TLR diversity across the east coast of Australia using 413 re-sequenced genomes at 30 × coverage. We identified 45 single-nucleotide polymorphisms (SNP) leading to 51 alleles within ten TLR genes. Our results show that the diversity of TLR genes in the koala forms four distinct genetic groups, which are consistent with the diversity of the koala major histocompatibility complex (MHC), another key immune gene family. The bioinformatics approach presented here has broad applicability to other threatened species with existing genomic resources.

## Introduction

The koala (*Phascolarctos cinereus*) is an arboreal marsupial native to Australia and distributed across eastern Australia and into the south-east of South Australia. However, koala populations have experienced significant declines across much of their natural range, with some areas seeing reductions of up to 80% over recent decades (Melzer et al. 2000). These declines are particularly pronounced in regions like New South Wales and Queensland, while other areas show more moderate decreases, leading to conservation concerns and efforts to protect the remaining populations and restore habitats. Koala populations have declined due to a number of threats, including habitat loss and fragmentation, climate change, bushfires, vehicle collisions, and disease (Lunney et al. 2014; Melzer et al. 2000). The two main diseases threats faced by koalas are *Chlamydia* and koala retroviruses (KoRV) (McCallum et al. 2018). *Chlamydia* is a bacterial infection that can cause reproductive problems, urinary tract infections, and ocular diseases, exerting considerable pressure on koala populations (Polkinghorne et al. 2013). KoRVs are a group of retroviruses that can infect by compromising immune function, affecting reproductive health, altering the koala genome, and potentially contributing to population decline (Denner and Young 2013).

The prevalence of chlamydial and KoRV infections varies between koala populations. Some individuals can successfully resolve the infection, while others cannot (Polkinghorne et al. 2013). *Chlamydia* infections are highly variable across regions, with some southern Australian populations, such as those on certain islands, showing little to no infection, while infection rates can range from 72 to 100% in northern populations (Polkinghorne et al. 2013). Importantly, not all koalas infected with *Chlamydia* develop disease symptoms; some individuals can successfully clear the infection, while others cannot, leading to varying levels of disease manifestation (Patterson et al. 2015). The prevalence of KoRV also differs across regions. KoRV has been detected in Queensland (QLD), most areas of New South Wales (NSW), and in approximately 72.2% of individual koalas assessed in mainland Victoria (VIC), compared to 26.6% on four Victorian islands (Simmons et al. 2012). Some southern populations remain KoRV-free, highlighting the uneven distribution of the virus (Stoye 2006).

Genetic diversity within immune genes plays a crucial role in maintaining the health and resilience of wildlife populations by influencing their susceptibility, or resistance, to infectious diseases. Research has shown a significant relationship between MHC diversity and infectious diseases in koalas (Lau et al. 2014; Quigley and Timms 2020; Robbins et al. 2020; Silver et al. 2022). A number of studies in humans and mice have found a correlation between toll-like receptor variation and susceptibility to invasive pathogens (Kesh et al. 2005; Misch and Hawn 2008; Moens et al. 2007; Netea et al. 2012), providing context for understanding similar patterns in wildlife. However, there have been no studies investigating the diversity of innate immune genes across the distribution of koalas.

Toll-like receptor (TLR) genes are an important innate immune gene family which recognise and binds to pathogens. The TLR molecule is composed of three domains: the extracellular binding domain, the transmembrane domain, and the intracellular toll/interleukin 1 (TIR) signalling domain (Yilmaz et al. 2005). The transmembrane and TIR signalling domains are highly conserved with the majority of variation in the extracellular binding domain, which comes into contact with antigens (Werling et al. 2009). The binding domain has a leucine-rich repeat pattern and is therefore also known as the leucine-rich repeat domain (LRR) (Alcaide and Edwards 2011). Inserts of leucine amino acids within the LRR domain may affect pathogen recognition (Offord et al. 2010), and single-nucleotide polymorphisms (SNPs) within this region can also affect the binding affinity of TLRs (Keestra et al. 2008). In contrast, the TIR signalling domain is largely conserved across the TLR family and phylogenetically among related species (Beutler and Rehli 2002). Koalas, like other marsupials, have ten TLRs (TLR2 to TLR10 and TLR13), each crucial for pathogen detection. Studies on *Chlamydia* infections in humans have shown that TLR2 and TLR4 recognise chlamydial components such as lipopolysaccharides (LPS) and lipoproteins and that TLR9 plays a role in promoting Th1 immune responses during these infections (Verweij et al. 2016; Yang and Joyee 2008). Furthermore, TLR3 and TLR7-9 are involved in recognising viral components, which may be important for detecting KoRV and triggering an antiviral immune response (Kayesh et al. 2021).

Genetic variation in TLR genes between individuals can lead to differences in their ability to recognise and respond to infectious diseases. Variation in TLR genes could result in variable levels of pathogen-associated molecular patterns (PAMPs) binding to *Chlamydia* causing variable immune responses to infection. Human studies have revealed that genetic variants in the TLR1, TLR2, TLR4, and TLR9 genes can increase inflammation and are associated with the risk of chlamydial infection and the development of pelvic inflammatory diseases, raising questions about whether genetic variation at these loci can serve as a biomarker of chlamydial infection (Taylor et al. 2012, 2013; Verweij et al. 2016). Most immunological studies, conducted primarily on mouse models, have shown that TLR2 and TLR4 activation and signalling strongly influence the elimination of chlamydial infection and the prevention of immunopathological sequelae (Beckett et al. 2012; Rodriguez et al. 2006). Our aim is to investigate whether similar patterns occur in wildlife. By characterising the diversity of TLRs in koala populations we aim to improve our understanding of the potential relationship between susceptibility to *Chlamydia* infection and TLR variations.

Recent studies advocate the use of genome re-sequencing to identify the diversity of immune genes in wildlife (Hogg et al. 2022), especially for species with low genome-wide diversity (Farquharson et al. 2021; Morris et al. 2015). Peel and colleagues emphasise the importance of long reads and scaffolding technologies for accurate annotation of immune genes due to their highly repetitive nature (Peel et al. 2022a). Re-sequenced genomes have been used successfully to characterise TLR diversity in endangered bird species (Magid et al. 2022) and across different cattle breeds (Novák et al. 2017). Here, we used a dataset of 413 re-sequenced koala genomes to characterise koala TLR diversity across the east coast of Australia. Previous work has described five primary genetic clusters with genetic diversity decreasing on a cline from northern QLD to VIC (McLennan et al. 2024). Ongoing research has also investigated MHC diversity in these koala populations, revealing a similar subdivision into five distinct groups (Silver et al. 2024). This study aims to characterise the diversity of SNPs within TLR genes in koalas to identify variations between populations. The results will provide a valuable resource for future research, particularly for understanding the potential association between TLR diversity and susceptibility to disease among koalas.

## Methods

### Study populations

**Fig. 1:**
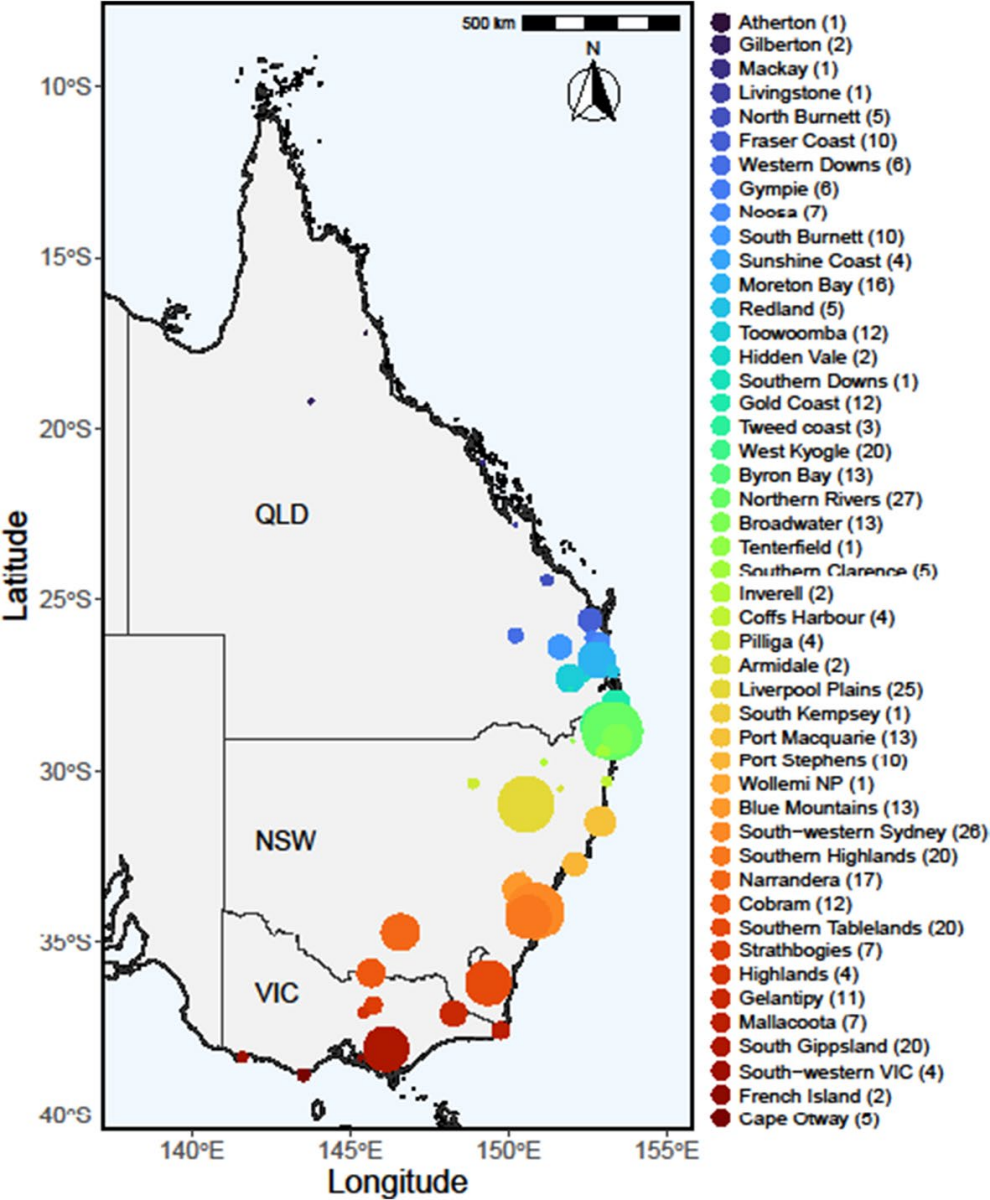
Sample distribution from Queensland, NSW, and Victoria. The colours of each spot represent different populations, which are divided into five genetic clusters as determined by McLennan et al. (in press)

We used short read sequence data for 413 wild koalas generated from previous work (Hogg et al. 2023) and aligned to the koala reference genome (NCBI: phaCin_unsw_v4.1) (Johnson et al. 2018). These koala samples were collected from northern Queensland to Victoria; no disease status data for either *chlamydia* or koala retrovirus were available for these samples. Based on the inferred population structure (McLennan et al. 2024), the 413 koalas were divided into five genomic clusters, including northern QLD (N_QLD), southeast QLD, and northern NSW (SEQLD_FNNSW), mid-coast NSW (M_NSW), southern NSW (S_NSW), and Victoria (VIC) (Fig. 1).

### TLR characterisation

#### Identifying candidate SNPs

To identify candidate single nucleotide polymorphisms (SNPs) within koala TLR genes, we used the “blastn” tool within BLAST v + searches (Altschul et al. 1990) with the manually annotated koala TLR reference sequences as queries (Peel et al. 2022b), and default parameters against the koala reference genome (phaCin_unsw_v4.1) (Johnson et al. 2018). Top blast hits for each TLR gene were checked for open reading frames and start/stop codons in IGV v2.17.0 (Robinson et al. 2011). Once the exonic regions of TLR were identified within the genome, variants falling exonic regions of TLR genes were filtered with VCFtools v0.1.14 and BCFtools v1.3.1 (Danecek et al. 2011). Variants were removed with minor allele frequency ≥ 0.05, Phred-score > 20, max-missingness = 0.9, minimum depth > 5, minGQ > 10, and maximum depth < 200. Additionally, the BCFtools v1.3.1 (Danecek et al. 2011) was used to remove sites with stand-bias phred-score < 60. SnpEff v5.2 (Cingolani et al. 2012) was used to characterise SNPs against koala genome annotation (Johnson et al. 2018) as non-synonymous or synonymous in exons.

#### TLR protein analysis and SNP location prediction

Two prediction tools, LRRfinder v4.0 (Offord et al. 2010) and SMART v9.0 (Letunic et al. 2021), were used to examine TLR protein sequences. LRRfinder utilises BLAST-based alignment to compare an input protein sequence to an existing database of TLRs, focussing on predicting potential LRR regions, which are highly variable and can influence the binding specificity and affinity of TLRs, specialising in detecting and analysing LRR motifs essential for pathogen recognition (Keestra et al. 2008; Offord et al. 2010). The second tool, SMART, uses Markov models to identify protein domains within TLRs by calculating the expected value for each domain found in the sequences, including the leucine-rich repeat (LRR), leucine-rich repeat N-terminal (LRRNT), leucine-rich repeat C-terminal (LRRCT), transmembrane (Trans), and intracellular toll-interleukin-1 receptor (TIR) domains (Letunic et al. 2021). The LRR, LRRNT, and LRRCT are crucial for the recognition and binding of pathogens, while the Trans domain is essential for the localisation of TLRs to the cell membrane. The TIR domain is critical for initiating intracellular signalling pathways in response to pathogen recognition. Variations in the LRR, LRRNT, LRRCT, and Trans domains may affect pathogen recognition, whereas variations in the TIR domain may impact the activation of immune responses (Mukherjee et al. 2019).

#### Diversity analysis

A consensus fasta file for each TLR gene in each individual was used to estimate diversity statistics using DNAsp v6.12.03 (Rozas et al. 2017). Individual genotype fasta files for koalas across TLR genes were created using VCFtools v0.1.14 and FastaAlternateReferenceMaker within GATK v4.2 (McKenna et al. 2010). The resulting files were imported into DNAsp v6.12.03 (Rozas et al. 2017) and analysed to calculate allele diversity, nucleotide diversity (*π*), and Tajima’s *D*. To compare koala TLR diversity with other species, we analysed the number of SNPs and alleles in two endangered species, the Tasmanian devil (*Sarcophilus harrisii*) and New Zealand Robin (*Petroica australis*), and two widespread species, the pig (*Sus scrofa*) and mockingbird (*Mimus polyglottos*). To assess genetic differentiation, between koala regions, we performed a principal coordination analysis (PCoA) based on TLR allele variants using Adegenet (Jombart 2008) in R v4.2.1. A Mantel test was performed to examine the correlation between genetic and geographic distances between different regions using the dartR package for R (Gruber et al. 2018).

## Results

We used 413 wild koalas from across the species range and grouped them into five known genomic clusters from northern Queensland (N_QLD; *N* = 87), southeast Queensland and northern New South Wales (SEQLD_NNSW; *N* = 114), mid-coast NSW (M_NSW; *N* = 63), southern NSW (S_NSW; *N* = 72), and Victoria (VIC; *N* = 77) (Fig. 1).

We were able to annotate all ten TLR genes in the 413 genomes (Table 1). In total, 45 SNPs were identified across the ten TLR genes, resulting in 51 alleles (Table 1). TLR1/6like was monomorphic, while the remaining nine TLRs were polymorphic. The number of SNPs observed in the nine polymorphic TLRs ranged from 3 to 11, and the number of alleles ranged from 3 to 15 alleles (Table 1). All polymorphic TLR genes contained at least one non-synonymous SNP except TLR2. The most variation was identified in TLR7 exhibited with 11 SNPs and 15 alleles. Tajima’s *D*-test investigated natural selection across different koala TLRs. Tajima’s *D* was not significant for most loci, except for TLR4, TLR5, and TLR9. TLR4 and TLR5 both showed potential deviations from the neutral evolution, whilst TLR9 was highly significant suggesting a significant departure from neutral evolution (Table 1). Out of the 45 SNPs, 28 SNPs were synonymous, and 17 SNPs were non-synonymous. Out of the 17 nonsynonymous SNPs, 7 were present across all 5 genomic clusters (Table 2). TLR7 showed the highest diversity with 11 SNPs (3 were non-synonymous), followed by TLR4 with 8 SNPs (3 non-synonymous), and both TLR5 and TLR10 with 5 SNPs each, having 2 and 3 non-synonymous SNPs, respectively. Thirteen of the nonsynonymous SNPs were located in the LRR domain, and 12 of them caused a physicochemical change due to the change in the identity of the amino acid (Table 2). These alterations are significant because they can affect the protein’s structure and its interactions with other molecules, potentially impacting the immune response.

**Table 1:** Genomic coordinates and variation in 10 TLR genes across 413 koalas

Gene	Scaffold	Strand	Start position	End position	SNPs (d*S*:d*N*)	No. alleles	Allelic diversity	*π*	Tajima’s *D*
TLR1/6like	MSTS0100003.1	+	11,877,704	11,880,122	0	1			
TLR2	MSTS01000056.1	+	7,981,884	7,984,236	3 (3:0)	5	0.4944	0.00041	1.82424
TLR3	MSTS01000124.1	−	160,216	160,445	3 (1:2)	3	0.2873	0.00018	0.28831
TLR4	MSTS01000040.1	−	4,959,426	4,961,655	8 (5:3)	10	0.8097	0.00107	2.27579*
TLR5	MSTS01000013.1	+	14,858,499	14,861,079	5 (3:2)	6	0.7168	0.00066	2.43424*
TLR7	MSTS01000122.1	+	1,447,018	1,450,159	11 (8:3)	10	0.7632	0.00098	1.86298
TLR8	MSTS01000122.1	+	1,496,962	1,500,084	3 (2:1)	4	0.6159	0.00033	1.99386
TLR9	MSTS01000216.1	−	372,491	375,570	3 (2:1)	5	0.748	1.99386	2.95088**
TLR10	MSTS01000003.1	+	11,925,125	11,927,487	5 (2:3)	5	0.6602	0.0006	1.75237
TLR13	MSTS01000154.1	−	6,155,624	6,158,487	4 (2:2)	3	0.4071	0.00041	1.73905
Total					45 (28:17)	51			

*dS* number of synonymous substitutions, *dN* number of non-synonymous substitutions, *π* nucleotide diversity

^*^P < 0.05; **P < 0.01

**Table 2:** Amino acid analysis of non-synonymous SNPs within TLR genes of the five genomic clusters

TLR	Binding specificity	AA site	TLR region	AA change	Physicochemical change	Population
TLR 3	dsRNA binding	305	LRR11	G/V	None	N_QLD, SEQLD_NNSW, M_NSW
		756	TIR	D/N	Charge/polar	N_QLD, SEQLD_NNSW, M_NSW
TLR4	LPS	341	LRR16	S/Y	Polar	N_QLD, M_NSW, S_NSW, VIC
		575	Trans	M/V	None	N_QLD, SEQLD_NNSW, M_NSW, VIC
		696	TIR	R/Q	None/polar	All 5 genomic clusters
TLR5	Flagellin	179	LRR6	Q/R	Polar/charge	All 5 genomic clusters
		584	LRRCT	Q/E	Polar/charge	N_QLD, SEQLD_NNSW, M_NSW, S_NSW
TLR7	Viruses	2	Signal	R/Q	None/polar	N_QLD, SEQLD_NNSW, M_NSW, S_NSW
		137	LRR4	E/K	Charge	N_QLD, SEQLD_NNSW, M_NSW, VIC
		665	LRR22	E/G	Charge/none	All 5 genomic clusters
TLR8	Viruses	454	LRR15	T/K	Polar/charge	All 5 genomic clusters
TLR9	Viruses	254	LRR8	A/V	None	All 5 genomic clusters
TLR10	Lipopeptide	164	LRR5	C/R	None/charge	All 5 genomic clusters
		213	LRR7	A/T	None/polar	SEQLD_NNSW, S_NSW, VIC
		464	LRR17	I/T	None/polar	SEQLD_NNSW, S_NSW, VIC
TLR13	Bacterial RNA	446	LRR17	S/C	Polar/none	SEQLD_NNSW, M_NSW, S_NSW, VIC
		708	LRRCT	R/S	None/polar	All 5 genomic clusters

In comparison to other species, our findings indicate that koalas have a higher overall diversity of TLR genes than the Tasmanian devil and the New Zealand Robin, but a lower diversity than pigs and mockingbirds (Table 3).

**Table 3:** Comparison of TLR diversity in species

Species	Genes	Samples	SNPs	Alleles	References
Koala	TLR1/6like, TLR2-5, TLR7-10, TLR13	413	48	51	This study
Tasmanian devil	TLR1/6like, TLR2-5, TLR7-10, TLR14	25–853	9	15	Farquharson et al. (2022)
New Zealand Robin	TLR2A, TLR2B, TLR3-5, TLR7	17–24	15	16	Grueber et al. (2012)
Pig	TLR4, TLR5	139	35	53	Palermo et al. (2009); Yang et al. (2013)
Mockingbirds	TLR1B, TLR4, and TLR15	212–228		81	Vlček et al. (2023)

Koalas have 51 alleles in ten genes, whereas pigs have 53 alleles in two genes

**Fig. 2:**
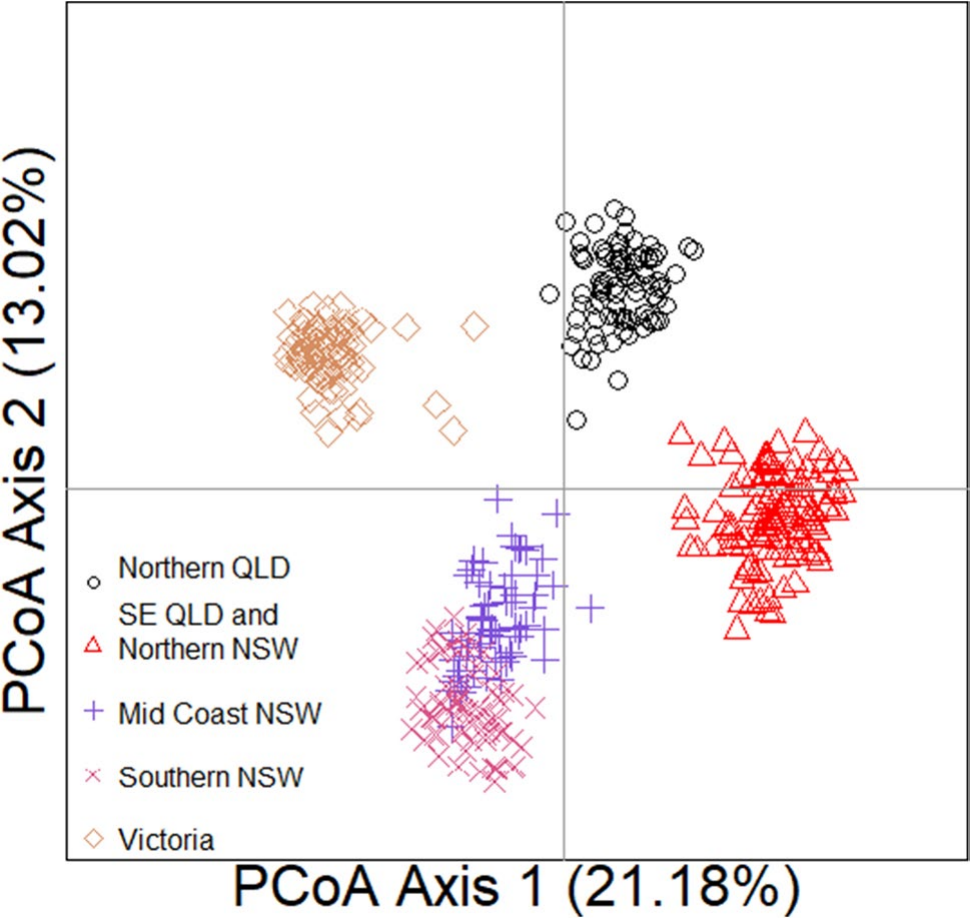
Genetic clustering of principal components of toll-like receptor variants in 413 wild koalas, with individuals coloured according to the region from which they originate, representing five previously identified wild koala genomic clusters

The PCoA revealed four relatively distinct genetic clusters with samples from northern QLD, southeast QLD and northern NSW, with the mid-coast NSW and southern NSW clustering together, and VIC (Fig. 2). Individuals from southeast QLD and far north NSW have the highest number of TLR alleles with 46, followed by the mid-coast NSW with 44 and northern QLD with 43. Southern NSW has 34 alleles, and VIC has the least, with 29 alleles (Table 4). Therefore, there are more alleles observed in the northern part of the koala’s range compared to the southern regions. Although the Mantel test was not significant, notable trends were noted between geographical distance and genetic distance (*r* = 0.6209, *p* = 0.0583).

**Table 4:** Number of alleles between genomic clusters

		No. of alleles in each genomic cluster				
Gene	No. alleles	N_QLD	SEQLD_NNSW	M_NSW	S_NSW	VIC
TLR2	5	5	5	5	3	3
TLR3	3	3	3	3	1	2
TLR4	10	8	8	10	6	6
TLR5	6	5	6	5	6	4
TLR7	10	8	8	8(A9)	5	3
TLR8	4	4	4	3	2	3
TLR9	5	5	5	5	4	3
TLR10	5	3	4	3	4(A4)	3
TLR13	3	2	3	2	3	2
Total	51	43	46	44	34	29

Two alleles were present in only one genomic cluster. TLR7 allele 9 (A9) and TLR10 allele 4 (A4) have only been found in M_NSW and S_NSW, respectively

## Discussion

This study used a bioinformatic approach to characterise the diversity of koala TLR genes using whole genome re-sequencing data. Analysing immune gene diversity through re-sequenced genomes is both cost-effective and efficient compared to traditional sequencing methods such as Sanger sequencing and first-generation sequencing. We examined the diversity in 10 TLR genes in 413 wild koalas from 50 different locations spanning the entire east coast of Australia, representing nearly the entire range of koalas. In total, we identified 45 SNPs leading to 51 alleles; of these, 17 SNPs led to non-synonymous variations. We have identified more TLR alleles compared to previous studies performed using amplicon sequencing of TLRs within koalas (Cui et al. 2015b).

We predicted that 12 of the 17 non-synonymous SNPs could lead to a physicochemical change in the koala. However, we do not have any phenotype data associated with this study to confirm these predictions. The predicted physicochemical changes could potentially affect protein–protein interactions, signal transduction, and downstream immune responses mediated by TLR. A previous study has suggested that significant changes in charge and hydrophobicity can influence the ligand-binding properties of TLR4 in birds (Velová et al. 2018). In koalas, physicochemical changes are also predicted in the TIR domains of TLR3 and TLR4. Alterations in the TIR domain could impact the ability of TLRs to detect pathogens and initiate appropriate immune responses (Kang and Lee 2011). This could affect an individual’s susceptibility to infectious diseases, inflammatory responses, or autoimmune conditions. To confirm our predictions and link these SNPs to specific health conditions, we need to collect both disease metadata and phenotypic data. These data would show how these SNPs correlate with health conditions and traits providing a greater understanding of disease distribution across the koalas’ range.

We identified 3 non-synonymous SNPs, located within the peptide-binding regions of TLR3, TLR5, and TLR13, determined by alignment analysis. These SNPs have the potential to induce structural modifications in the respective TLRs, thus influencing their functionality. In particular, the koala variant TLR3 G305V shares a location with the human TLR3 c.908 T > C (p.Phe303Ser), a known mutation associated with signalling defects through disruption of receptor-ligand interaction (Hidaka et al. 2006). The variant TLR5 Q179R in koalas is located within the LRR6 domain. Variations within the LRR6 may influence the overall conformation of TLR5, potentially affecting its ability to interact with flagellin (Song et al. 2017). That is, changes within this region might alter the binding affinity or stability of the flagellin-TLR5 complex. The variant TLR13 R708S of the koala, located in the C-terminal region of the LRR (LRRCT), corresponds to the C-terminal side of the peptide binding region (Song et al. 2015). These genetic variations potentially induce alterations in TLR structure and function, thereby affecting inflammatory signalling pathways and immune activation (Werling et al. 2009). The variants TLR5 Q179R and TLR13 R708S also lead to physicochemical change, which is expected to negatively affect the function of proteins (Song et al. 2015, 2017). These genetic changes may weaken the individual’s ability to fight infections or properly manage inflammation, leading to potential health issues.

Our study identified TLR4, TLR5, TLR7, and TLR10 as the most variable TLR genes in koala populations. We found additional diversity not detected in a previous study that was the first to characterise TLR diversity in koalas (Cui et al. 2015b). The higher diversity we observed here was likely because we examined TLR diversity across the koala geographic range using over 400 individuals, compared to 20 animals previously (Cui et al. 2015b), and used a re-sequenced genome dataset with higher SNP discovery. Using whole-genome data has allowed us to capture a more comprehensive picture of TLR polymorphisms, suggesting potential regional variation in TLR diversity. TLR diversity is crucial for pathogen recognition (Miller et al. 2005; Werling et al. 2009) and is shaped by positive selection, affecting both viral and nonviral TLRs (Areal et al. 2011). Previous research has demonstrated that KoRV infection influences TLR expression patterns in peripheral blood mononuclear cells (PBMCs) of koalas, with up-regulation of TLR4-7 and TLR10 observed in koalas infected with endogenous and exogenous KoRV subtypes compared to those infected only with KoRV-A (Kayesh et al. 2021). This suggests that different KoRV subtypes affect the expression levels of specific TLRs. Our study focused on identifying SNP variations in TLRs across koala populations, and integrating these findings with earlier expression data (Kayesh et al. 2021) highlights the possibility that TLR genetic variation might correlate with differential responses to KoRV. Further studies that have disease metadata should focus on the relationship between TLR variants, expression, and KoRV infection.

The diversity of TLR in koalas exceeds that of species facing population bottlenecks and decli ning wild populations, such as the Tasmanian devil (Cui et al. 2015a; Farquharson et al. 2022) and the New Zealand Robin (Grueber et al. 2012). It is important to note that our study used whole genomes, providing a more comprehensive assessment of genetic diversity compared to other studies that have used targeted specific gene sequences like target capture or amplicon sequencing. However, the TLR diversity in koalas is less than what is seen in species such as pigs (Palermo et al. 2009; Yang et al. 2013) and mockingbirds (Vlček et al. 2023). TLR diversity reductions in koalas could compromise their ability to combat pathogens and adapt to environmental changes. With fewer TLR variants available, their immune system may struggle to recognise and respond to a broad range of pathogens, leading to increased vulnerability to infections and disease.

Unlike previous studies that indicated five genomic clusters for koalas using genome-wide SNPs, our results showed only four primary groups of koalas (McLennan et al. 2024). This suggests that TLR alleles and SNPs may reflect different aspects of genetic diversity and evolutionary pressures in the species, which is to be expected. Koalas from mid-coast and southern NSW had notable overlap with their TLR alleles, compared to what was described using genome-wide SNPs (McLennan et al. 2024); however, there was some evidence of sub-structuring. However, our clustering results are consistent with the diversity of MHC koala previously described (Silver et al. 2024), indicating that selective pressures of disease are likely driving the observed variation in koala immune gene diversity. Further study using whole-genome resequencing coupled with known disease data and phenotypic data will improve our understanding of this relationship, this work is currently underway. Importantly, our findings highlight the need to preserve genetic diversity within and among koala genomic clusters. Reduced genetic diversity could impair their adaptability and disease resistance, making it crucial to protect and maintain varied genetic profiles to support their long-term survival. (Hogg et al. 2023; Lott et al. 2022).

The current diversity of TLRs in wild koalas is probably shaped by past population bottlenecks caused by historical hunting, habitat loss, disease, and environmental events (Martin and Handasyde 1999; Menkhorst 2008; Phillips 2000). Although bottlenecks generally reduce genetic diversity, selective pressures from diseases, such as *Chlamydia* and KoRV, have potentially influenced the retention and prevalence of certain TLR alleles, thereby affecting the overall immune competence of koala populations. Comparatively, in the European rabbit (*Oryctolagus cuniculus*), TLR3 shows high diversity in wild populations, possibly due to pathogen exposure (Abrantes et al. 2013). Similarly, in the bottlenecked population of the Seychelles warbler (*Acrocephalus sechellensis*), the diversity of TLRs is influenced by genetic drift and potential balancing selection. Genetic drift has reduced the variation of the TLR gene, but some functional variation remains, particularly at the TLR15 locus, due to possibly balancing selection (Gilroy et al. 2017). In the case of koalas, directional selection might also play a role. Southern koala populations, which have low genetic diversity, show low incidences of *Chlamydia* and KoRV (Polkinghorne et al. 2013; Simmons et al. 2012). This pattern suggests that specific TLR variants may be favored in these populations, potentially conferring resistance to these diseases. Further investigation is needed to confirm this hypothesis and better understand the genetic basis of disease resistance in these populations.

Although not significant, the Mantel test showed a trend between geographic distance and genetic distance. This suggests that the observed genetic differences are likely influenced by the geographic distribution of the genomic clusters, with closer clusters being more genetically similar and distant ones more genetically distinct. Although the genome-wide analysis found a statistically significant relationship between genetic and geographic distance (McLennan et al. 2024), our non-significant result is likely the result of the conserved nature of immune genes (Gonzalez‐Quevedo et al. 2015). The variation we do observe possibly reflects local adaptation to their respective environments (Taylor et al. 2011). Genetic variation in TLR could be driven by differences in disease pressures or environmental conditions across geographical gradients, with local ecological niches fostering genetic differences that accumulate over time (Netea et al. 2012). Research on genetic variation in TLR genes suggests that differences in disease pressures and environmental conditions across geographical gradients can drive this variation (Ferwerda et al. 2007; Grueber et al. 2013). Furthermore, historical events, such as population expansions, contractions, or founder effects, may have left lasting imprints on the genetic structure of these koala populations (Lee et al. 2010a, 2010b). Further research into the specific mechanisms driving this correlation would provide valuable insights into the dynamics of the koala population and inform conservation practices aimed at preserving its genetic diversity and combatting disease.

In conclusion, a whole genome re-sequenced dataset efficiently characterises koala TLR gene diversity, providing cost-effectiveness and applicability to non-model species. Koalas exhibit higher TLR diversity than species with population bottlenecks but lower than general widespread species. The diversity of TLR of the koala grouped into four clusters similar to what has been observed in the MHC genes. This study highlights the potential for broad applications of re-sequenced genomes in the conservation of wildlife, emphasising its utility not only for koalas but also for a wide range of non-model species.

## Data Availability

Raw whole genome re-sequences are available to download from The National Center for Biotechnology Information (NCBI) under BioProject PRJNA940526. All metadata for the koala genomes is available via Zenodo (10.5281/zenodo.13777157).
